# Statistical Analysis of Multisensory and Text-Derived Representations on Concept Learning

**DOI:** 10.3389/fncom.2022.861265

**Published:** 2022-04-27

**Authors:** Yuwei Wang, Yi Zeng

**Affiliations:** ^1^Research Center for Brain-Inspired Intelligence, Institute of Automation, Chinese Academy of Sciences, Beijing, China; ^2^School of Artificial Intelligence, University of Chinese Academy of Sciences, Beijing, China; ^3^Center for Excellence in Brain Science and Intelligence Technology, Chinese Academy of Sciences, Shanghai, China; ^4^National Laboratory of Pattern Recognition, Institute of Automation, Chinese Academy of Sciences, Beijing, China

**Keywords:** concept learning, multisensory representations, text-derived representations, representational similarity analysis, concreteness

## Abstract

When learning concepts, cognitive psychology research has revealed that there are two types of concept representations in the human brain: language-derived codes and sensory-derived codes. For the objective of human-like artificial intelligence, we expect to provide multisensory and text-derived representations for concepts in AI systems. Psychologists and computer scientists have published lots of datasets for the two kinds of representations, but as far as we know, no systematic work exits to analyze them together. We do a statistical study on them in this work. We want to know if multisensory vectors and text-derived vectors reflect conceptual understanding and if they are complementary in terms of cognition. Four experiments are presented in this work, all focused on multisensory representations labeled by psychologists and text-derived representations generated by computer scientists for concept learning, and the results demonstrate that (1) for the same concept, both forms of representations can properly reflect the concept, but (2) the representational similarity analysis findings reveal that the two types of representations are significantly different, (3) as the concreteness of the concept grows larger, the multisensory representation of the concept becomes closer to human beings than the text-derived representation, and (4) we verified that combining the two improves the concept representation.

## 1. Introduction

One key element of cognition is concept learning, or the capacity to identify commonalities and emphasize contrasts across a set of related events in order to develop structured knowledge (Roshan et al., 2001). The current availability of brain imaging techniques has raised curiosity on how concepts are encoded in the brain. Huth et al. (2016) mapped semantic selectivity across the cortex using voxel-wise modeling of whole-brain blood-oxygen-level-dependent (BOLD) responses data collected while subjects listened to hours of narrative stories. They built a comprehensive semantic atlas that demonstrates that the distribution of semantically selective regions is symmetrical throughout the two cerebral hemispheres, with nice individual consistency. According to neurocognitive studies, the semantic system is topologically divided into three brain modules: multimodal experiential representation, language-supported representation, and semantic control, leading to the proposal of a tri-network model of semantic processing (Xu et al., 2017). Psychological studies have shown that the human brain has (at least) two types of object knowledge representations: one based on sensory-derived codes and one based on language/cognitive-derived codes, both supported by separate brain systems. It is difficult to distinguish the contribution of them in human subjects (Wang et al., 2020).

From the perspective of quantification, recent concept learning researches also concentrated on two aspects: multisensory representations and text-derived representations (Davis and Yee, 2021). Multisensory representations are based on embodied theory, which emphasis that meaning is grounded in our sensory, perceptual, motor and experiences with the world (Barsalou, 1999). While text-derived representations are relied on the distributional hypothesis, which states that the similarity between two concepts is rooted in the similarity of their linguistic contexts (Harris, 1954).

On the one hand, multisensory representations are basically obtained from psychology experiments. By asking participants how strongly they experienced a particular concept by hearing, tasting, feeling through touch, smelling, and seeing, Lynott and Connell proposed modality exclusivity norms for 423 adjective concepts (Lynott and Connell, 2009) and 400 nominal concepts (Lynott and Connell, 2013) on strength of association with each of the five primary sensory modalities. Analogous vectors are now available in a variety of languages, such as French (Bonin et al., 2014), Spanish (Díez-Álamo et al., 2017), Dutch (Speed and Majid, 2017), Russian (Miklashevsky, 2017), Chinese (Chen et al., 2019), and Italian (Vergallito et al., 2020). Lynott et al. (2019) published Lancaster Sensorimotor Norms, which expanded the norms to 11 dimensions, including six perceptual modalities (auditory, gustatory, haptic, interoceptive, olfactory, visual) and five action effectors (foot/leg, hand/arm, head, mouth, torso). With 39,707 psycholinguistic concepts, this dataset is the largest ever. Based on more recent neurobiological evidences, Binder et al. (2016) established a set of brain-based componential semantic representation with 65 experiential characteristics, spanning sensory, motor, spatial, temporal, affective, social, and cognitive experiences. This dataset includes 535 concepts and performs well when distinguishing a priori conceptual categories and capturing semantic similarity.

On the other hand, text-derived representations are generated from computational linguistics. Word2vec and GloVe are two representative models for transforming semantic and syntactic information of words into dense vectors. Word2vec (Mikolov et al., 2013) comprises two models: continuous bag of words model that learns to predict the current word given the context, and skip-gram model that learns to predict context words given the current word. GloVe (Pennington et al., 2014) is a specific weighted least squares model that trains on word-word co-occurrence counts matrix which integrates global matrix factorization and local context information. They are the most significant and often used text-derived representations. They've recently gotten a lot of attention for their impressive results in a variety of natural language processing tasks.

**Figure 1:**
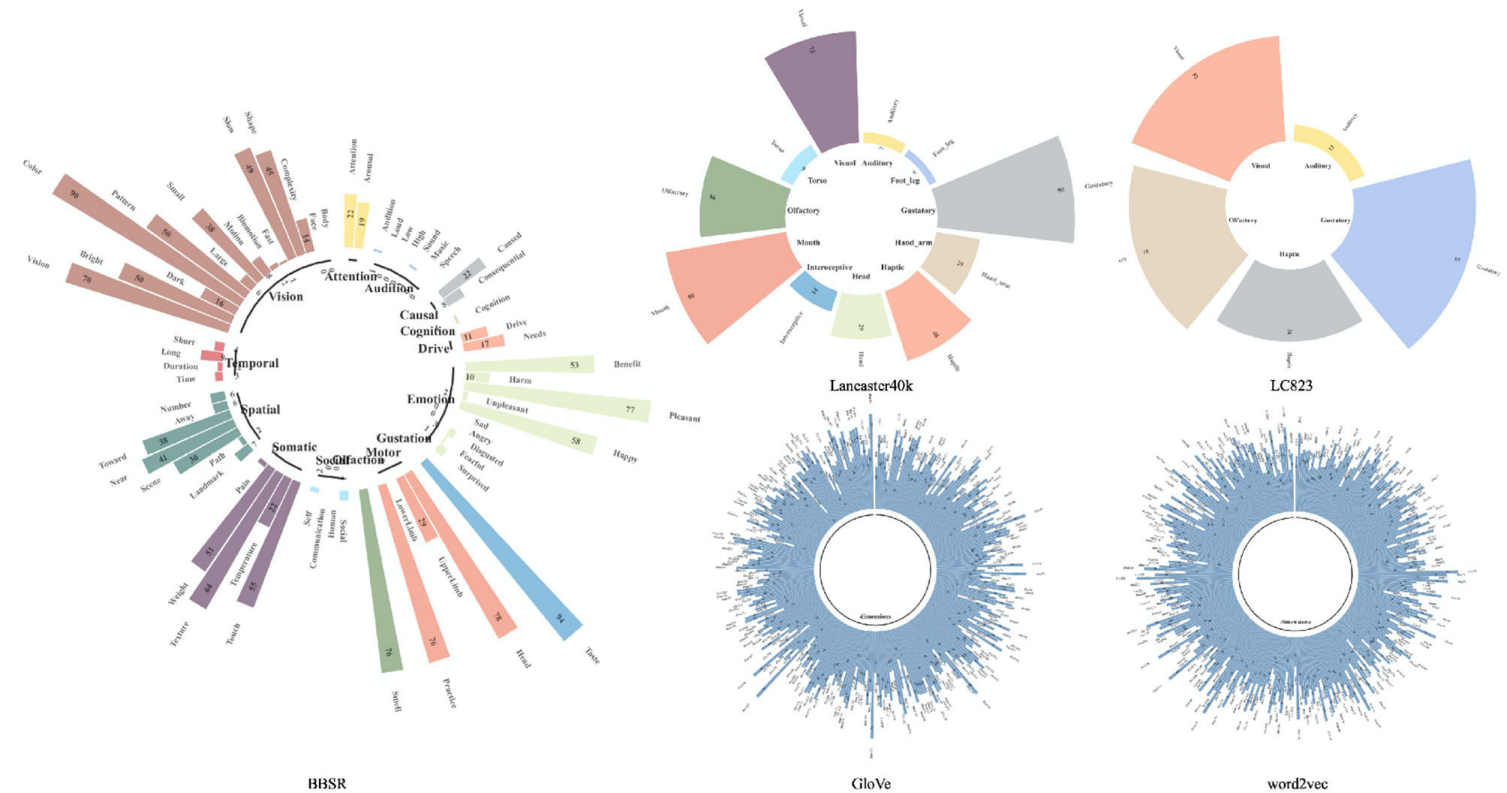
A demo representation of the concept “Honey” in the concept representation dataset mentioned in this article. The circular bar of the same concept “honey” is shown here in LC823 (Lynott and Connell, 2009, 2013), Lancaster40k (Lynott et al., 2019), BBSR (Binder et al., 2016), word2vec (Mikolov et al., 2013), and GloVe (Pennington et al., 2014). It is obvious that the multisensory vectors have good interpretability, as each dimension has clear information referring to it, whereas we are unsure what each dimension in the text-derived vectors represents.

Figure 1 demonstrates the same concept “honey” in the two types of datasets. For multisensory representations, each dimension represents the perceptual strength while for text-derived representations the dimension information is like a “black box”, with weak interpretability. Despite the fact that there has been a lot of research on how to integrate the two types of vectors for improved concept learning (Hill and Korhonen, 2014; Hill et al., 2014a; Kiela and Bottou, 2014; Silberer and Lapata, 2014; Collell et al., 2017; Wang et al., 2018), there has been no systematic comparison between the vectors of different sources as far as we know.

To verify whether these concept representation datasets provide a solid foundation for human-like intelligence, the quantitative analysis of the two types of representations will be carried out through four experiments. In what follows, we describe four experiments implicating statistical analysis of multisensory and text-derived representations on concept learning. The first experiment focuses on k nearest neighbors for the same concept from multisensory and text-derived perspectives, the second one concentrates on representational similarity analysis on two types of vectors, the third one emphasizes on the influence of concept's concreteness for multisensory and text-derived vectors, and the fourth one proves that the combination of the two improves the concept representation.

## 2. Multisensory and Text-Derived Representations: A Micro Analysis

Similar concepts will share similar features, which is an essential characteristic of concept learning in cognitive activities. In this section, we try to investigate whether similar concepts are also similar in multisensory and text-derived representation spaces.

### 2.1. The Criterion

Semantic feature norms are a way of displaying concepts by utilizing normalized and systematic feature descriptions which reflect the human understanding of the concepts. These semantic norms shed light on a variety of human behaviors including concept perception, categorization, and semantic memory (McRae et al., 2005). For example, the features of the concept “celery” are “*is_green”, “a_vegetable”, “has_stalks”, “is_stringy”, “has_leaves”, “is_long”, “has_fibre”, “is_edible”, “is_crunchy”, “eaten_in_salads”, “eaten_with_dips”, “tastes_bland”, “tastes_good”, “grows_in_gardens”*, and “*is_nutritious”*. The intersection and difference of semantic feature norms relate to the similarities and contrasts between concepts. For example, the shared features for “car” and “scooter” are “*has_wheels”, “used_for_transportation”, “has_an_engine”, “is_fast”*, and “*a_vehicle”*. While the unique features of “car” are “*has_4_wheels”, “has_doors”, “has_a_steering_wheel”* and the unique features of “scooter” are “*has_2_wheels”, “has_handle_bars”, “used_with_helmets”*, showing their difference.

The primary objective of obtaining semantic feature norms is to create interpretable conceptual representations that can be used to evaluate theories of semantic representation and computation. The most influential work in this respect is **McRae** semantic feature norms, which is proposed by McRae et al. (2005). They not only presented 541 concepts with their feature norms, but also suggested a methodological framework to generate them. **CSLB** (Centre for Speech, Language and the Brain) is another semantic feature norms dataset which is comparable with McRae (Devereux et al., 2014). They improved the procedure of feature normalization and feature filtering, collecting 866 concepts. This article takes McRae and CSLB as the criterion for human conceptual cognition to explore how multisensory and text-derived representations are linked to human cognition.

### 2.2. The Methods

In this study, the multisensory vectors are represented by Lancaster40k^1^ (Lynott et al., 2019) and BBSR (brain-based componential semantic representation)^2^ (Binder et al., 2016), whereas text-derived vectors are represented by word2vec^3^ (Mikolov et al., 2013) and Glove^4^ (Pennington et al., 2014).

Firstly, we get all the similar concepts for each concept in multisensory and text-derived concept representation datasets respectively (measured *via* cosine similarity), sort them by similarity, and record their rankings. Next, in the semantic feature norms datasets such as McRae and CSLB, we select the k closest neighbors of each concept (the similarity is determined by counting the number of features that overlap), and find the their rankings' median in each representation dataset separately. The smaller the ranking, the closer the representations are to human perception. As Table 1 shows, in the criterion dataset McRae, the closest neighbor (*k* = 1) for the concept “accordion” is “saxophone”. The “Reasons” show the overlapped features of the concept pair. The similarity rankings of “saxophone” for the concept “accordion” in multisensory datasets BBSR and Lancaster40k and text-derived datasets GloVe and word2vec are 5, 48, 5, 4 separately. Finally, we obtain the average value for each type of representations. As k varies, we can draw a scatter plot and perform linear fitting.

**Table 1:** The closest neighbor (*k* = 1) demo in McRae.

**Concept**	**Closest neighbor**	**Reasons**	**Ranking in BBSR**	**Ranking in Lancaster40k**	**Ranking in gloVe**	**Ranking in word2vec**
Accordion	Saxophone	A musical instrument; has keys; requires air; produces music;	5	48	5	4
Blueberry	Plum	A fruit; is round; is small; tastes sweet; is edible; is juicy; eaten in jams; tastes good	3	59	12	69
Magazine	Book	Has pages; has words in it; made of article; has pictures	1	3	1	1
Pumpkin	Tomato	Has seeds; is round a fruit; a vegetable; grows on vines	2	8	6	2
Truck	Van	Has wheels; has 4 wheels; used for cargo; a vehicle; is large; used for transportation; requires gasoline; has an engine	1	2	19	1

*The reasons come from the overlapped features of each concept pair*.

### 2.3. Results and Analysis

**Figure 2:**
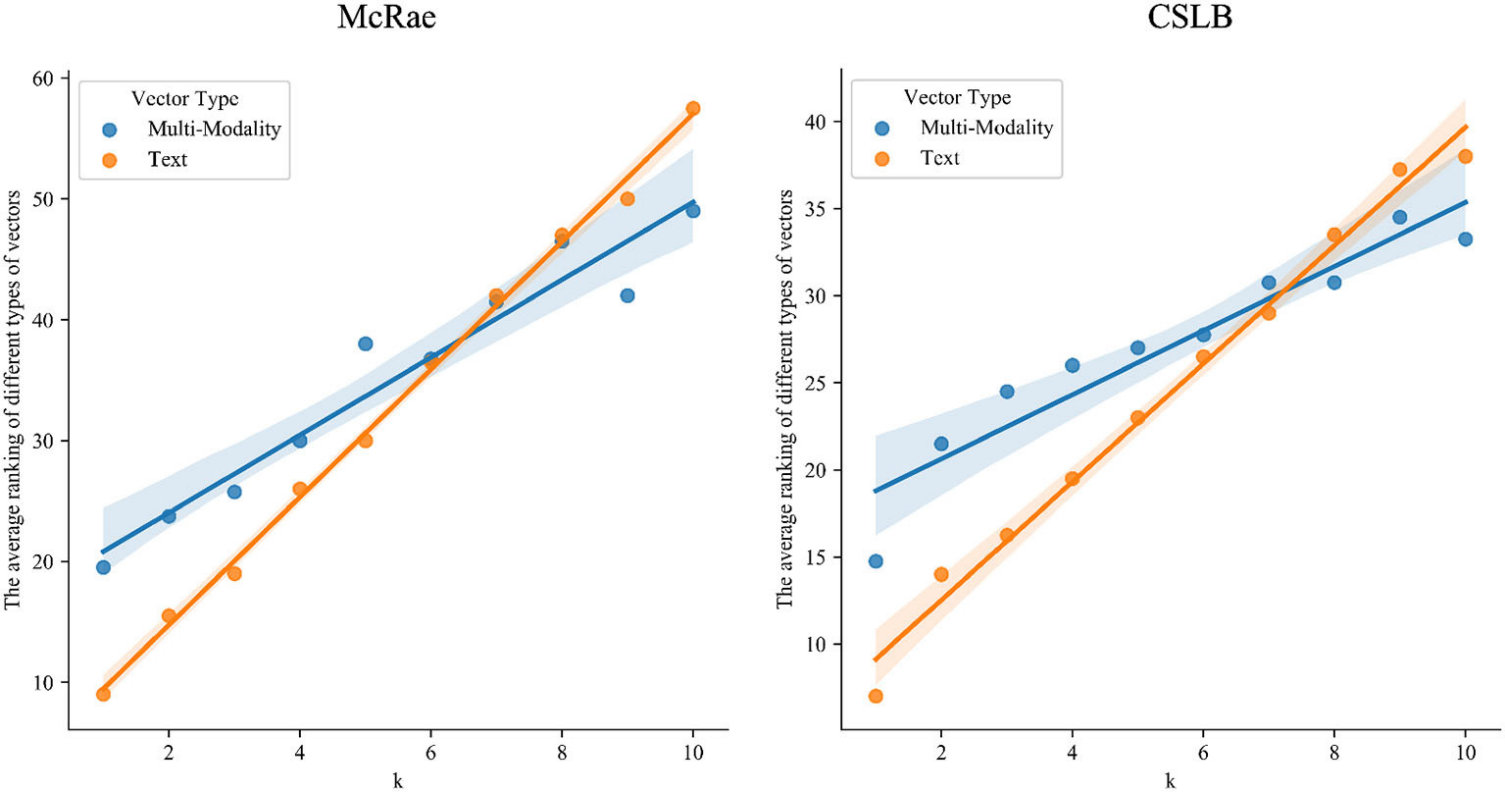
The median of k closest neighbors' ranking in McRae and CSLB.

Table 2 and Figure 2 illustrate the findings. The results demonstrates that: (1) Either multisensory or text-derived vectors exhibit remarkable linearity as k varies, suggesting that they both accurately reflect the essence of the concept, which is identical to human beings. This means that similar concepts in the space of human cognition are also similar in the spaces of both multisensory and text-derived representations (2) The results of both types of representations show the same tendency, though with difference slope. For smaller values of *k*, the multisensory representations show better performance, while the text vector-based representations are closer to human for larger values of *k*. (3) Detailedly, text-derived vectors which are trained based on large-scale corpus are more stable, but less interpretable. We can easily locate similar concepts for each concept, but we have no idea what each dimension means or why they are related. Multisensory vectors, on the other hand, are based on psychological labeling and have high interpretability. We know what each dimension represents, whereas the dimension information for text-derived representations is unclear. We can identify which modality is responsible for similarity between the two concepts. However, there is a larger variance different multisensory vectors. This is probably due to the fact that Lancaster40k has just 6 dimensions and therefore has limited representational capacity, but BBSR, with 65 dimensions, can better deal with such a situation.

**Table 2:** Median rankings of k closest neighbors.

**Median of rankings**	**McRae**				**CSLB**			
	***k* = 1**	***k* = 3**	***k* = 5**	***k* = 10**	***k* = 1**	***k* = 3**	***k* = 5**	***k* = 10**
BBSR	2	4.5	8	13	2	4	6	10.5
Lancaster40k	37	47	68	85	27.5	45	48	56
Average	19.5	25.75	38	49	14.75	24.5	27	33.25
GloVe	9	19	32	59	6	16	22	36
w2v	9	19	28	56	8	16.5	24	40
Average	9	19	30	57.5	7	16.25	23	38

## 3. Multisensory and Text-Derived Representations: A Macro Analysis

The above experiment shows that both kinds of the vectors mirror the concept itself, thus is there an inherent relationship between multisensory and text-derived representations from a macro perspective? To explore this, we use representational similarity analysis (RSA) to evaluate distinct vectors and detect the relationship between them *via* hierarchical clustering.

### 3.1. Representational Similarity Analysis

In the field of cognitive neuroscience, RSA is a computational approach that bridges the divides between brain-activity measurement, behavioral measurement, and computer modeling (Kriegeskorte et al., 2008). RSA is a data-analytical framework for analyzing how neural activity is quantitatively related to each other, as well as to computational theory and behavior, using representational dissimilarity matrices (RDMs), which characterize the information carried by a given representation in a brain or model. RSA allows us to compare representations inside a brain or model, across brain and behavioral data, and between humans and species (Nili et al., 2014). RSA reflects the degree of similarity between two representation spaces. In this study, we utilize RSA to examine the connection between the two types of representations using their typical vectors.

### 3.2. The Method

Besides BBSR, Lancaster40k, word2vec, and GloVe, we also introduce **LC823** as a multisensory typical dataset that combines Lynott and Connell's data from 2009^5^ (Lynott and Connell, 2009) and 2012^6^ (Lynott and Connell, 2013). For the sake of consistency, we will focus on the effects of five types of senses in this experiment: vision, touch, sound, smell, and taste. We use the first five dimensions of Lancaster40k, while we normalize the data and use the average value of the sub-dimensions corresponding to these five senses in BBSR.

**Figure 3:**
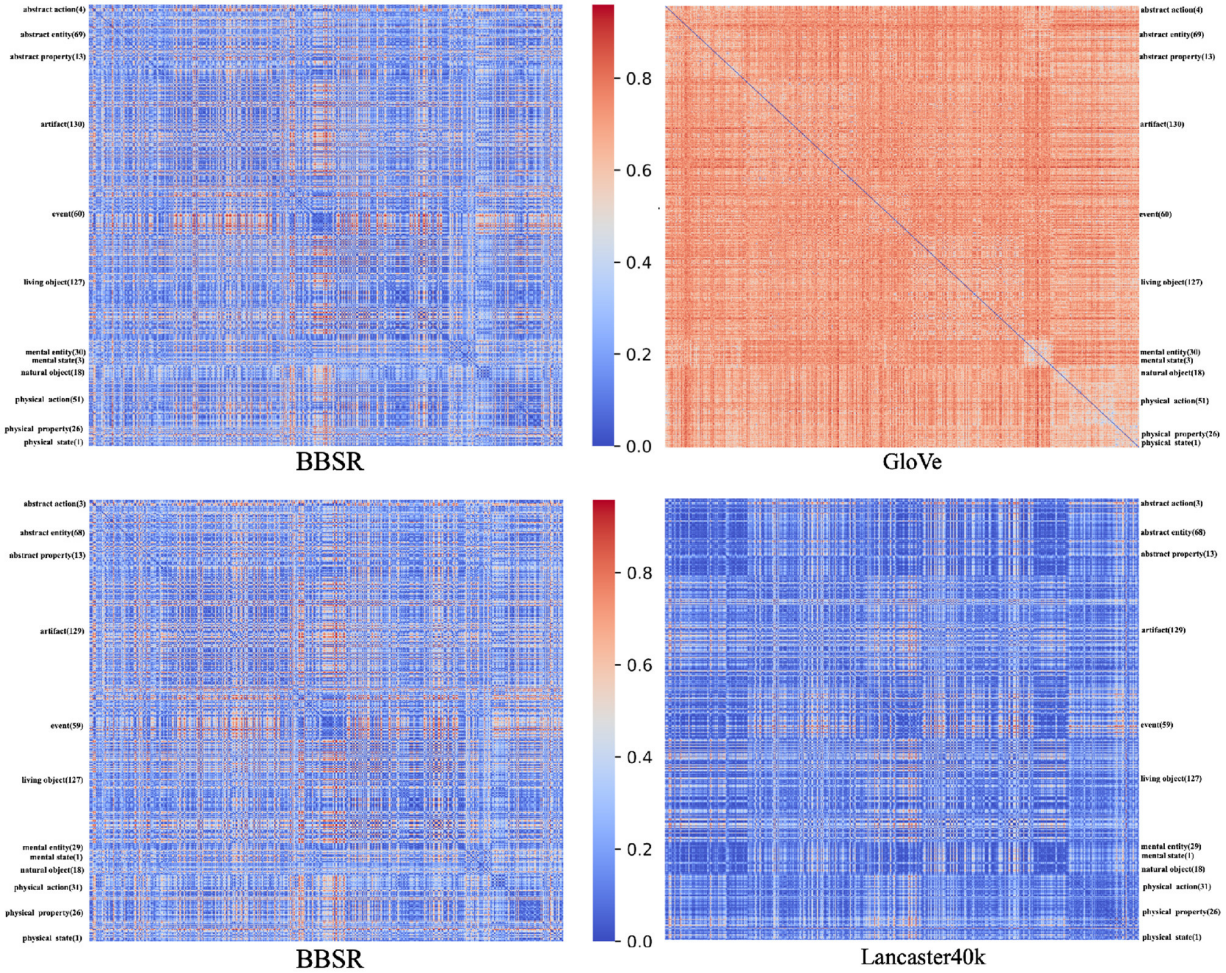
Representational dissimilarity matrices demonstration. The RDMs between BBSR and GloVe are shown above, while the RDMs between BBSR and Lancaster40k are shown below. For each matrix, all concepts are displayed in order of category, with category categorization based on BBSR.

For these five datasets of different sources, we analyze each two as a pair separately. We obtain the overlapped concepts from the corresponding datasets in this pair and construct RDMs using these concepts. RDM is symmetric about a diagonal of zeros, and each cell carries a score that indicates the difference between concept pairs. Additionally, the concepts in each of the two RDMs are presented in the same order. In this article, we use cosine distance to measure the dissimilarity. Figure 3 exhibits RDM demonstrations. The RDMs between BBSR and GloVe are shown above, while the RDMs between BBSR and Lancaster40k are shown below. For each matrix, all concepts are displayed in order of category, with category categorization based on BBSR.

**Figure 4:**
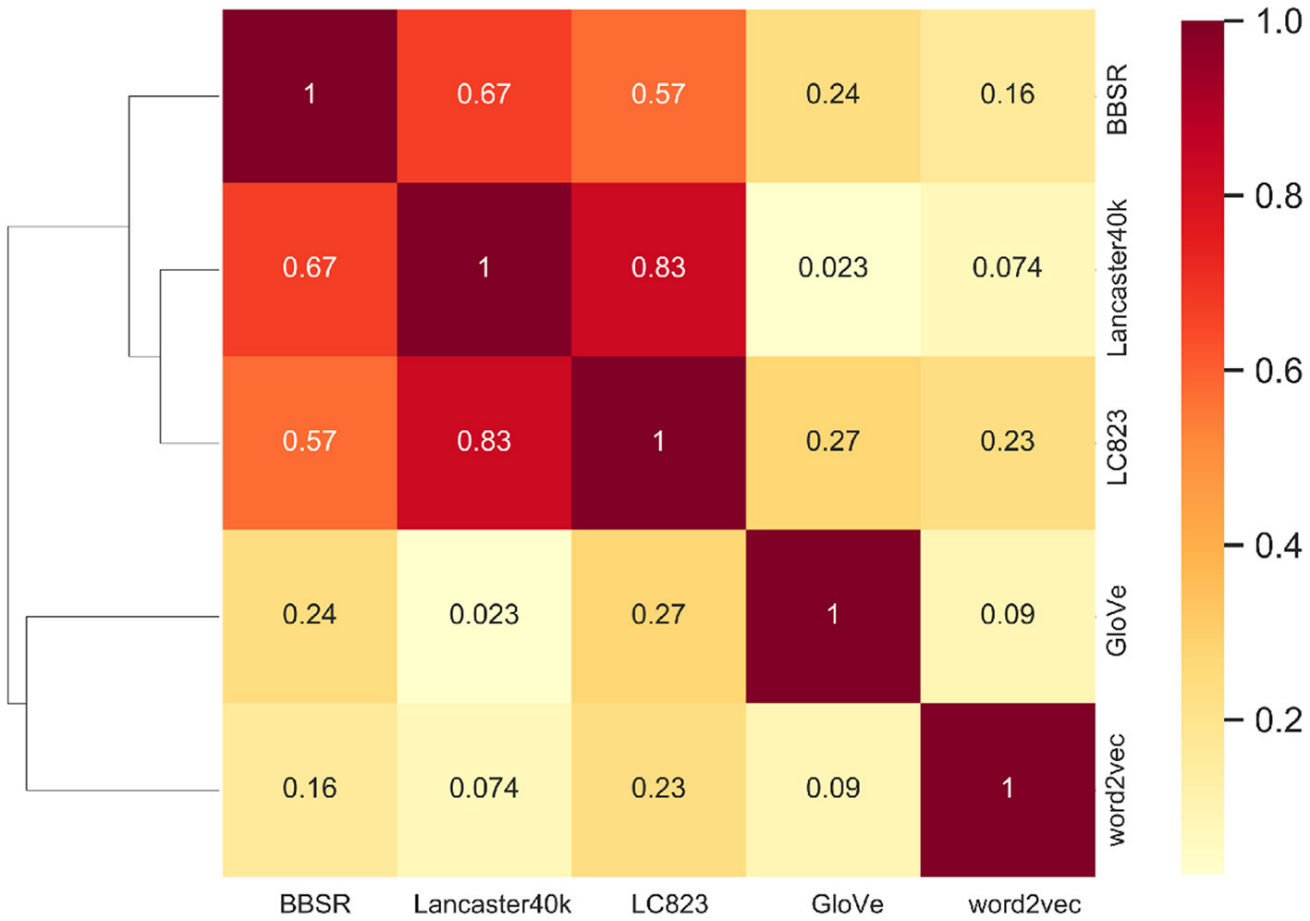
Representational similarity analysis on two types of representations. The matching strength between multisensory and text-derived vectors is shown in the heatmap on the right side of the figure, and the hierarchical clustering structure on the left is constructed using the Matching Strength and Euclidean distance.

The Spearman correlation between the upper diagonal portions of the two RDMs is referred to as “Matching Strength”, which evaluates the macroscopic match between two representation spaces in terms of the degree of comprehension about the same concept. The Matching Strength between each representation dataset pair is shown in Figure 4. For example, the Matching Strength between BBSR and Lancaster40k is 0.67 while the Matching Strength for BBSR and word2vec is 0.16. We perform an unsupervised clustering analysis based on the these Matching Strength results. Euclidean distance is used and the hierarchical clustering structure is constructed.

### 3.3. Results and Analysis

The within-category correlation for the same concept is higher for the same type of vector representation, whereas the correlation between different types of representations is lower, as shown in Figure 4 for the RSA and clustering findings. *Via* unsupervised learning, the data points are divided into two parts, which are nicely related to multisensory and text-derived representations. Between the two types of representations, there is a clear distinction.

This is probably due to the fact that the two types of representation vectors are based on different theoretical foundations and data sources: multisensory representations are based on embody theory, whereas text-derived representations are based on distributed theory; multisensory representations are primarily derived from psychologists' research, whereas text-derived representations are primarily obtained from computer scientists' training with large-scale data.

When combined with the micro analysis results in the above section, we could draw the interesting conclusion that there is no significant difference in the effect of the two distinct types of representations for the same concept, but the original aim and source of the two representations differ. This supports the findings of Wang et al. (2020), who claim that the human brain has (at least) two types of concept representations. It suggests that the available multisensory and text-derived representation spaces are very identical to the human brain's representation space.

## 4. The Gap Analysis

So the question arises, what causes the gap between these two types of vectors? In this experiment, we will explore the sensitivity of the two types of representations to the concepts' concreteness, a quantifiable property of concepts.

### 4.1. Concreteness

Concreteness is a property of the concept in psychological study that reflects the degree to which something may be experienced *via* our senses. The concept with a higher concreteness rating relates to something that exists in reality, while the concept with a lower concreteness rating refers to something that you cannot directly experience *via* your senses or actions. The recognition and processing of concrete concepts is usually faster than that of abstract concepts (Schwanenflugel et al., 1988), while the emotional valence of abstract concepts is higher than that of concrete ones, resulting in a residual latency advantage for abstract words (Kousta et al., 2011). Many datasets involving concreteness exist in the field of cognitive linguistics. **Concreteness40k**, proposed by Brysbaert et al. (2013) is the biggest concreteness rating dataset, with 37,058 English words and 2,896 two-word phrases gathered from over 4,000 people through a norming research that used internet crowdsourcing for data collecting. They utilize a 5-point scale that ranges from abstract to concrete. The **Glasgow** Norms are a another set of normative ratings for 5,553 concepts on nine psycholinguistic dimensions: arousal, valence, dominance, concreteness, imageability, familiarity, age of acquisition, semantic size, and gender association, and they are the most comprehensive psycholinguistic materials ever created (Scott et al., 2019). The Glasgow Norms' dimensions are all based on 7-point rating systems. For generality, in this study, we quantify the concreteness of the concepts separately using Concreteness40k and the concreteness part in Glasgow Norms.

### 4.2. Human-Like Concept Learning Metric

Most cognitive functions, such as categorization, memory, decision-making, and reasoning, are based on human similarity and relatedness judgments between concepts. As a result, there is a large collection of human-labeled measure datasets to evaluate the degree of human-likeness from the standpoint of concept similarity and concept relatedness, particularly in the domains of natural language processing (Lastra-Diaz et al., 2021). To assess how well each type of representation reflects human judgments, we compute Spearman correlations between model-based similarity and human assessments, as is customary. The larger the correlation coefficient, the more similar to human cognition, i.e., more human-like.

In this article, we evaluate the closeness of multisensory representations and text-derived representations to humans using multiple datasets such as Ag201 (Agirre, 2009), MC28 (Miller and Charles, 1991), MEN (Baroni et al., 2014), MT235, MT287 (Radinsky et al., 2011), MT771 (Halawi et al., 2012), PSfull (Pirró, 2009), Rel122 (Szumlanski et al., 2013), RG65 (Rubenstein and Goodenough, 1965), RW1401, RW2034 (Luong et al., 2013), SCWS1994 (Huang et al., 2012), SL111, SL222, SL665, SL999 (Hill et al., 2014b), SV3500 (Gerz et al., 2016), VS (Silberer and Lapata, 2014), WS353, WS353r, WS353s (Agirre et al., 2009), YP130 (Yang and Powers, 2006), as well as McRae and CSLB utilized in Experiment 1 (the cosine similarity of feature-based one-hot representations determines the rating for each concept pair).

### 4.3. The Method

Given the large number of measure datasets involved, BBSR and LC823 have limited concept tagged and the overlap with the measure datasets is small, therefore in this section we just utilize Lancaster40k as a representation of multisensory vectors, while GloVe and word2vec remain as text-derived representatives. In this experiment, we investigate the relationship between the concreteness of different concepts and the closeness of their representations to human beings for the two types of representations.

We get the associated concreteness for each concept pair *(concpet1, concept2)* in the measure dataset (if any concept in the pair cannot be mapped, the pair is ignored) and define their mean value as the pair's concreteness, $conc^{pair}=\frac{\left(conc^{concept1}+conc^{concept2}\right)}{2}$. We furthermore average the concreteness of all the pairs to obtain the concreteness of the whole measure dataset i.e., $conc^{dataset}=\frac{\underset{all\;pairs\;in\;the\;dataset}{\sum }conc^{pair}}{\#\;of\;the\;pairs}$. For each type of vectors, we calculate the closeness as described above for each measure dataset and obtain the Pearson correlation between closeness *clos*^*dataset*^ and measure dataset concreteness *conc*^*dataset*^.

### 4.4. Results and Analysis

**Figure 5:**
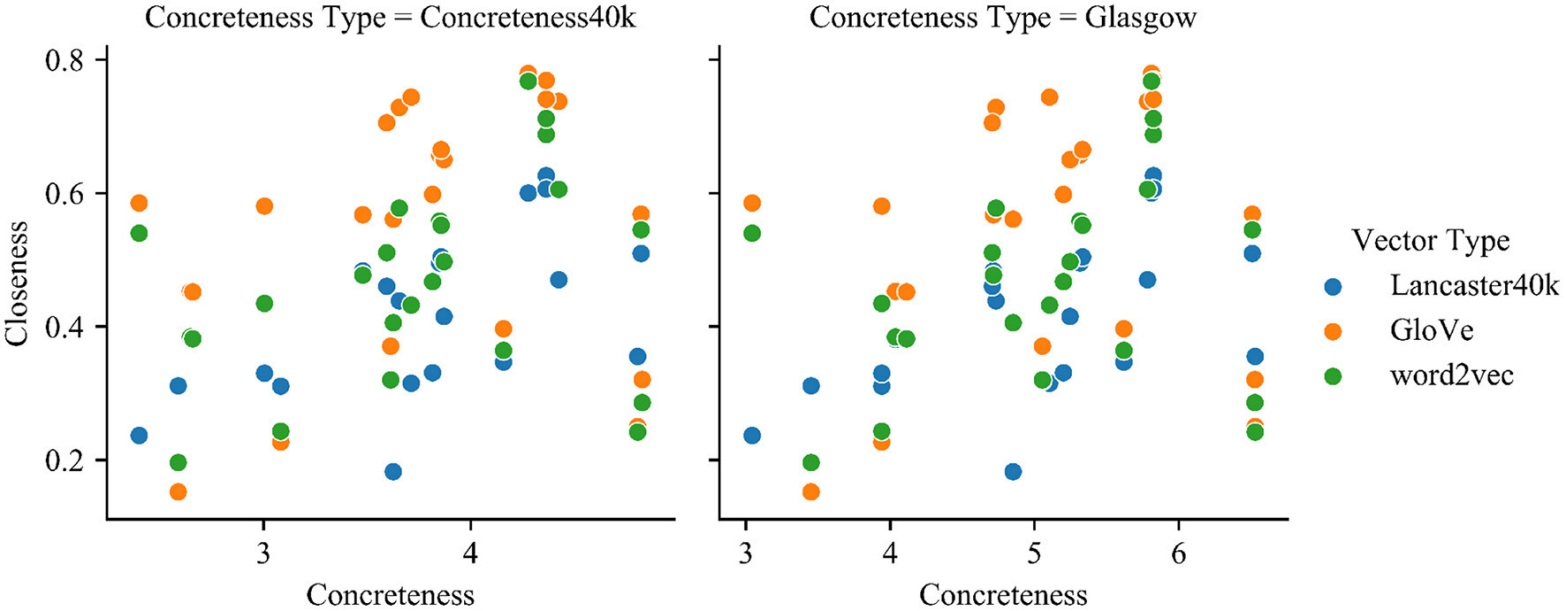
The relationship between concreteness and closeness for each type of vectors.

Figure 5 and Tables 3, 4 show that for the multisensory vectors, the association between the closeness and the concreteness of the concepts is stronger, showing that the introduction of multimodal information can better characterize the concept itself for concepts with larger concreteness. In contrast, the effect of the vector of text representations is less related to the concreteness of the concepts, and the distribution is more scattered, which may be related to the fact that the generation method is based on large-scale corpus training, and the acquisition of concepts is dependent on context or word frequency, as opposed to multisensory vectors, which take more into account the environment.

**Table 3:** Closeness analysis on human-like concept learning metrics.

		**Ag201**	**CSLB**	**MC28**	**McRae**	**MEN**	**MT235**	**MT287**	**MT771**	**PSfull**	**Rel122**	**RG65**	**RW1401**	**RW2034**	**SCWS1994**	**SL111**	**SL222**	**SL665**	**SL999**	**SV3500**	**VS**	**WS353**	**WS353r**	**WS353s**	**YP130**
Statistics	Number of pairs	201	85,407	28	47,448	3,000	235	287	771	65	65	1,401	2,034	122	1,994	111	222	665	999	3,500	7,576	353	252	203	130
	Number of concepts	275	638	37	541	751	405	499	1,113	48	48	2,145	2,951	240	1,706	107	170	749	1,028	827	502	437	346	277	147
	Lancaster40k (overlapped pairs)	195	67,191	28	38,992	2,944	170	162	754	65	101	65	897	926	1,809	111	222	665	999	3,487	6,816	330	235	195	130
	Lancaster40k (overlapped concepts)	265	565	37	490	742	294	287	1,094	48	199	48	1,400	1,444	1,550	107	170	749	1,028	824	477	410	326	265	147
	GloVe (overlapped pairs)	201	74,788	28	42,647	3,000	177	169	771	65	95	65	871	900	1850	111	222	665	999	3,498	7,507	334	238	196	126
	GloVe (overlapped concepts)	275	598	37	515	751	307	300	1,113	48	187	48	1,364	1,408	1,584	107	170	749	1,028	826	501	416	331	266	143
	word2vec (overlapped pairs)	200	76,616	28	42,396	2946	176	169	771	65	95	65	903	934	1,848	111	222	665	999	3,500	7,447	334	238	196	126
	word2vec (overlapped concepts)	273	605	37	513	747	305	300	1,113	48	187	48	1416	1,464	1,583	107	170	749	1,028	827	499	416	331	266	143
Concreteness	Concreteness40k	3.85	4.81	4.28	4.83	4.43	3.65	3.59	3.87	4.37	3.71	4.37	2.65	2.66	3.48	2.4	2.59	4.16	3.61	3.08	4.82	3.82	3.63	3.86	3
	GlasgowCNC	5.31	6.53	5.81	6.53	5.78	4.73	4.71	5.25	5.82	5.1	5.82	4.04	4.11	4.71	3.04	3.45	5.62	5.06	3.94	6.51	5.2	4.85	5.33	3.94
Closeness	Lancaster40k	0.5	0.36	0.6	0.32	0.47	0.44	0.46	0.41	0.63	0.32	0.61	0.38	0.38	0.48	0.24	0.31	0.35	0.32	0.31	0.51	0.33	0.18	0.5	0.33
	GloVe	0.66	0.25	0.78	0.32	0.74	0.73	0.71	0.65	0.74	0.74	0.77	0.45	0.45	0.57	0.59	0.15	0.4	0.37	0.23	0.57	0.6	0.56	0.67	0.58
	word2vec	0.56	0.24	0.77	0.29	0.61	0.58	0.51	0.5	0.69	0.43	0.71	0.38	0.38	0.48	0.54	0.2	0.36	0.32	0.24	0.55	0.47	0.41	0.55	0.43

**Table 4:** Correlation analysis on concreteness and closeness.

**Pearson correlation**	**Concreteness40k**	**GlasgowCNC**
Lancaster40k	0.465503079	0.474294653
GloVe	0.237656528	0.210263777
word2vec	0.303239538	0.271815609

## 5. The Combination

The previous three experiments show that for each concept, the multisensory and text-derived representation can both properly suit the concept and make the representation close to human. However, this does not imply that the representations of these two different types of sources are the same; on the contrary, there are considerable distinctions between them, particularly for concepts of varying concreteness, where various representations have different effects. With the development of NLP technology, text-derived representations based on large-scale corpus training have emerged, but most of them are based on pure text and do not include the influence of environmental and multisensory information.

Existing text-derived representation datasets are much larger in scale than multisensory representations, so current conceptual representations of AI systems are mostly dominated by text-derived representations. The preceding studies show that text-only derived representations bias human cognition for concepts with high concreteness, but multisensory representations are better at describing such concepts. These two kinds of codes are compatible in the human brain, and we intend to investigate whether the vectors of the two types of representations are also complimentary from a quantitative aspect. We also want to see if adding multisensory information to text-derived vectors helps to increase their representational capacity.

### 5.1. The Method

Lancaster40k and BBSR are still used as multisensory vectors, whereas GloVe and w2v are used as text-derived vectors in this experiment. This section focuses on the possibility of merging the two vectors rather than on how the two types of vectors should be merged to get the best outcomes, therefore the most naive merge method is chosen to for them. For each concept, we concatenate its multisensory vector and text-derived vector as the combined vector to represent it. The evaluation measure utilized in this section is still the Human-like Concept Learning Metric from the Gap Analysis part, and this part we only utilizes McRae and CSLB as measure datasets.

### 5.2. Results and Analysis

**Figure 6:**
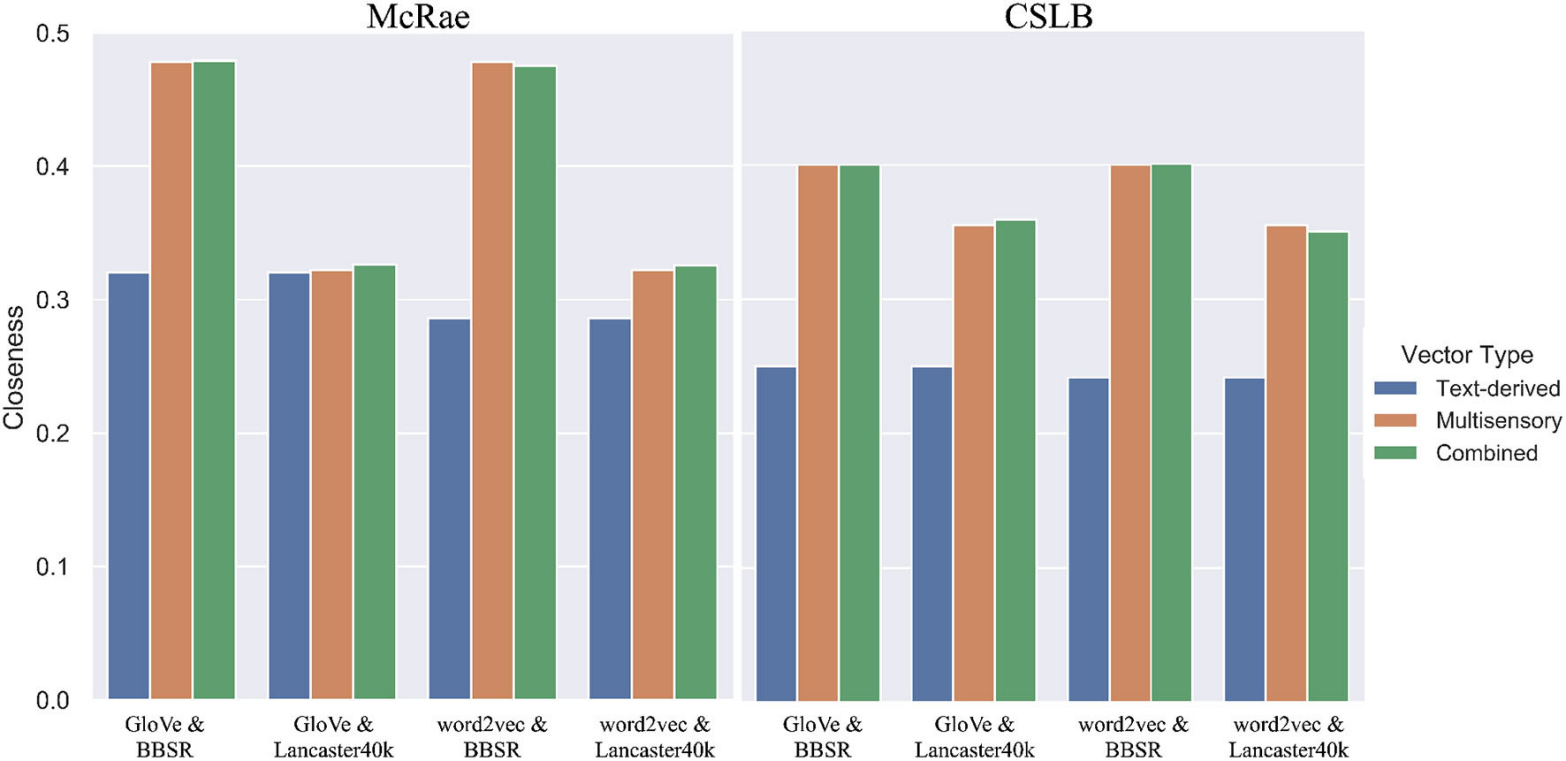
The closeness for multisensory vectors, text-derived vectors and their combination.

We concatenate two of the four multimodal or text vectors together and record their separate closeness as well as the combined closeness. As demonstrated in Figure 6, multisensory representations and text-derived representations are obviously complimentary. In each of the four combinations of the two measure datasets, all the fused vectors outperformed the text-derived vectors on their own. This implies that integrating multisensory vectors with text-derived vectors in AI systems could be beneficial. Six fused representations outperform non-fused representations in all eight scenarios, showing that the combination of direct connections improves concept learning and makes the representation closer to human cognition. However, this is not the case in all circumstances, suggesting that the way in which the two representations are integrated is worth further exploration.

## 6. Conclusion and Future Works

In this work, we perform four experiments for concept learning with multisensory and text-derived representations, analyze the similarities and differences between them, and prove that combining the two can improve concept representations. We verified, by means of quantitative analysis, that the available multisensory and text-derived representation datasets are in great agreement with cognitive findings. Combining the two types of vectors can well enhance the representational capabilities and help the development of human-like AI.

We utilize the two types of most typical vector datasets in all of the above tests. However, from the perspective of cognitive theory, these two representations still have a lot of issues to work out. The publicly accessible vector datasets for multisensory representation are based on psychologists' annotations, which are extremely interpretable but more “expensive”. Due to the limitations of annotation engineering and some rare or abstract concepts, the size of such concept vectors is difficult to scale up. On the other hand, we can collect textual corpus for almost no cost *via* web crawlers, databases, big data technologies, open source communities, and so on. With various text vector generation algorithms, we can extract concept or word vectors from the corpus.

Although these vectors can accurately capture the vector representation of the corpus domain and depict the similarity and relatedness of concepts, their interpretability is limited. We can't grasp the meaning of a single dimension since its value is derived by defining the loss function as well as the contextual relationship. Unlike multisensory representations, where they are apparent what make two concepts similar or not, for each dimension is perceptual strength related.

Although this text-based concept learning technique based on large-scale corpus training can deliver rapid and efficient text-based responses in some AI systems, it would be unable to include common sense information, making the system less human-like. Therefore, from an algorithmic standpoint, can we avoid the downsides of both while maximizing the benefits of both?

Aside from the aforementioned data acquisition issues, two forms of dimensional balancing issues are also worth investigating. Multisensory representations have modest dimensions, a few tens at most, but text-derived representations are relatively flexible, with approximately 300 being the most common. How to balance the two types of information from an algorithmic perspective remains to be explored. Additionally, despite the fact that the two kinds of representations are derived from different sources, one based on distributed theory and the other on embedding theory, it remains to be seen if there are explanatory and effective mapping models that may improve the scale of multisensory representation.

Furthermore, this research only proves in the most basic way that merging two distinct vectors can enhance the concept learning system. Current fusion techniques are mostly based on traditional machine learning technologies to design algorithms. Spiking neural networks are a variety of brain-like neural network algorithm that integrates temporal information, making them more human-like in terms of information computation and showing promise. It's also worth investigating whether using SNN to combine two vectors would yield better results. Even more importantly, how do humans fuse various types of idea representations in the brain, and do they fuse in the same manner for different types of concepts? There is still no conclusive answer. We're eager to see related research that will inspire us to produce meaningful algorithms.

## Data Availability Statement

The original contributions presented in the study are included in the article/supplementary material, further inquiries can be directed to the corresponding author/s.

## Author Contributions

YW and YZ designed the study, performed the experiments, and wrote the manuscript. All authors contributed to the article and approved the submitted version.

## Funding

This study was supported by the Strategic Priority Research Program of the Chinese Academy of Sciences (Grant No. XDB32070100).

## Conflict of Interest

The authors declare that the research was conducted in the absence of any commercial or financial relationships that could be construed as a potential conflict of interest.

## Publisher's Note

All claims expressed in this article are solely those of the authors and do not necessarily represent those of their affiliated organizations, or those of the publisher, the editors and the reviewers. Any product that may be evaluated in this article, or claim that may be made by its manufacturer, is not guaranteed or endorsed by the publisher.

## Footnotes

^1^ http://osf.io/7emr6/

^2^ http://www.neuro.mcw.edu/resources.html

^3^https://code.google.com/archive/p/word2vec/ the pre-trained vectors were trained on part of Google News dataset.

^4^https://nlp.stanford.edu/projects/glove/ the version of pre-trained text-derived vectors is Common Crawl (840B tokens, 2.2M vocab, cased, 300d vectors, 2.03 GB download).

^5^ https://link.springer.com/article/10.3758/BRM.41.2.558

^6^ https://link.springer.com/article/10.3758/s13428-012-0267-0
